# Solar Tracking Error Analysis of Fresnel Reflector

**DOI:** 10.1155/2014/834392

**Published:** 2014-05-06

**Authors:** Jiantao Zheng, Junjie Yan, Jie Pei, Guanjie Liu

**Affiliations:** ^1^School of Energy and Power Engineering, Xi'an Jiaotong University, Xi'an 710049, China; ^2^Huaneng Clean Energy Research Institute, Beijing 100098, China

## Abstract

Depending on the rotational structure of Fresnel reflector, the rotation angle of the mirror was deduced under the eccentric condition. By analyzing the influence of the sun tracking rotation angle error caused by main factors, the change rule and extent of the influence were revealed. It is concluded that the tracking errors caused by the difference between the rotation axis and true north meridian, at noon, were maximum under certain conditions and reduced at morning and afternoon gradually. The tracking error caused by other deviations such as rotating eccentric, latitude, and solar altitude was positive at morning, negative at afternoon, and zero at a certain moment of noon.

## 1. Introduction

Solar energy is plentiful and unlimited. Solar-photovoltaic power generation and solar thermal power generation are two basic solar-energy-utilization ways [1]. Owing to solar thermal power generation performing good stability, high reliability, long working time, and middle/high temperature steam provision, solar thermal power generation is widely focused in the energy field.

In 1960s, Giovanni Francica (Italy) firstly applied linear Fresnel reflector (LFR) for development of linear and two-axis tracking Fresnel steam generating system [2, 3]. From then on, lots of research was gradually carried out. In order to enhance the utilization of land area and reduce the shading created by adjacent reflectors, Mills and Morrison invented the multiabsorption towers technology based on compact linear Fresnel reflector which was suitable for large-scale solar thermal power plants [4, 5]. Mathur et al. proposed an optimal design of the width of primary mirror [6]. Sootha and Negi made optical design and focused characteristic research about linear concentrating technology without secondary reflectors [7]. Du et al. estimated the tracking and radiation for LFR system by increasing the spacing between the adjacent reflectors and got the best method for estimating height [8]. Pu and Xia designed a small linear concentrating test device, conducting the light tracing simulation and concentration research [9].

The above research is carried out to optimize the key components and eventually achieve the real-time sun tracking by the tracking program, so the concentrating system and tracking accuracy are key components to realize the high efficiency, high performance parameters in Fresnel solar thermal power generation system. There are many ways to realize the solar tracking. In the condition of low tracking accuracy requirement or reducing complexity and costs, open loop single axis tracking method can be used [10]. Du et al. resolved sun vector properly by using vector method, and then the tracking rotation angle was got after simple geometric derivation, which was applicable to linear Fresnel reflector without the eccentric structure [11].

This paper analyzes the main impact factors of the tracking accuracy of Fresnel solar reflectors and calculated the tracking error and its influence.

## 2. Fresnel Tracking System Error and Analysis

### 2.1. Fresnel Lens System Introduction

**Figure 1:**
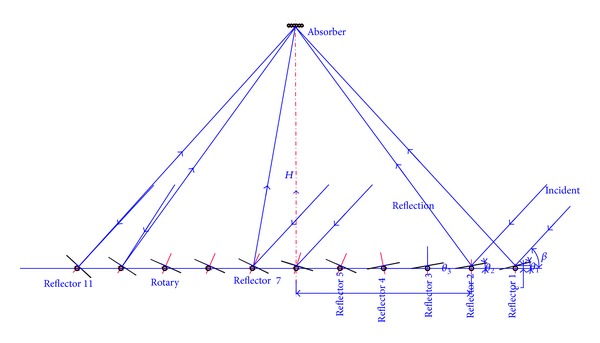
Diagram of the Fresnel solar reflection technology.

The LFR field which are comprise of 11 rows of flat mirrors can be imagined as a broken-up parabolic trough reflector by using linear segments to focus the radiation, as Figure 1 shows. The parabolic trough shape is simulated by several flat reflectors at different positions, in order to make the sunlight reflect to the receiver tubes fixed on the top of mirrors. Each mirror must tracks accurately, to ensure the virtual parabolic shape and keep the reflected solar radiation on the absorber all the time. Therefore, improving tracking accuracy plays an important role in promoting the collection efficiency [12].

The tracking error of Fresnel reflectors could be affected by time, mirror exocentric structure, collector orientation, height of absorbers, axis position, solar altitude angle, and so on. This paper analyzed the influence of these factors on the tracking error.

### 2.2. The Analysis of Tracking System Rotation Angle

**Figure 2:**
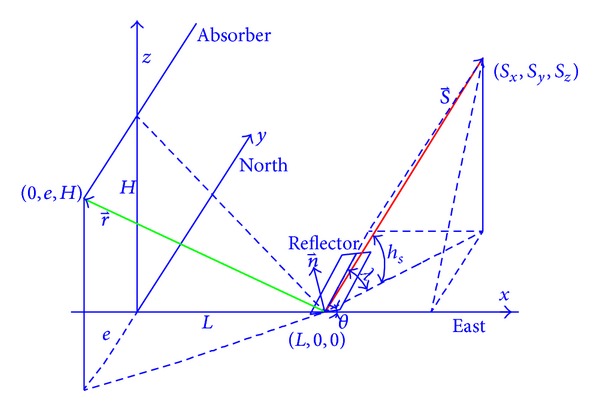
Diagram of ray reflection of Fresnel technology.

It is well known that in order to track the sun the exact position of sun is necessary. The main parameters of sun position are shown in Figure 2, which are sun declination angle *δ*, hour angle *ω*, elevation angle *θ*, geographic latitude *φ*, and longitude *ε*. The sunlight irradiates to point *P* on the surface of earth, which is determined by elevation angle *h*_*s*_ and azimuth angle *λ* of the tangent plane. The sun position in the tracking system is calculated by international SPA algorithm whose accuracy is 0.0003° [13, 14].

In Figure 2, the sunlight irradiates to the center of mirror (*L*, 0,0) at space vector of elevation angle *h*_*s*_ and azimuth angle *λ*; reflection light is vector $\overset{⃑}{r}$, and the normal vector of central point of mirror is $\overset{⃑}{n}$. The angle between mirror and horizontal *X*-axis direction is *θ*, and the location position of reflected rays on collector tubes is (0, *e*, *H*).

If the mirror rotation center is located on the center of mirror, one has the following:

(1) r⃑=(−LR,eR,HR),  R=L2+e2+H2,n⃑=(−sinθ,0,cos⁡θ),s⃑=(sx,sy,sz),s⃑×n⃑=|i⃑j⃑k⃑sxsysz−sinθ0cos⁡θ|=sycos⁡θi⃑−(sxcos⁡θ+szsinθ)j⃑+sysinθk⃑,n⃑×r−=|i⃑j⃑k⃑−sinθ0cos⁡θ−LReRHR|=−eRcos⁡θi⃑−(LRcos⁡θ−HRsinθ)j⃑−eRsinθk⃑.

According to the law of light reflection, it is known that

(2) $$\begin{array}{c} s_{y}\cos\theta =-\frac{e}{R}\cos\theta ,\\ s_{x}\cos\theta +s_{z}\sin\theta =\frac{L}{R}\cos\theta -\frac{H}{R}\sin\theta ,\\ s_{y}\sin\theta =-\frac{e}{R}\sin\theta . \end{array}$$

Then

(3) $$\begin{array}{c} \tan\theta =\frac{L/R-s_{x}}{H/R+s_{z}},\\ e=\frac{-s_{y}}{\sqrt{1-s_{y}^{2}}}\sqrt{L^{2}+H^{2}}. \end{array}$$

In the equation, *s*_*x*_, *s*_*y*_, *s*_*z*_ are the vector components of sun position along the *X*, *Y*, *Z* axis. The rotation angle of mirror *θ* and deviation distance e of reflected solar radiation in the direction of absorber can be calculated at a certain moment.

There are two rotation methods for mirror rotation: one is rotating around the geometric center of the mirror and the other is using the reflector supporting shaft as rotation center. This paper mainly analyzes the tracking error under the second condition.

When the rotation axis is not located in the center of mirror, *L* and *H* in formula (3) need to be replaced by *L*-*R* sin*θ* and *H*-*R*cos⁡⁡*θ*. Assuming that mirror width is 800 mm, spacing between adjacent reflectors is 200 mm, and focal length is 7 m, the tracking error under different deviation factors was obtained by computer program.

## 3. The Influence of Main Parameters Deviation on Tracking Accuracy

### 3.1. The Influence of Frame Eccentric Error on Tracking Accuracy

**Figure 3:**
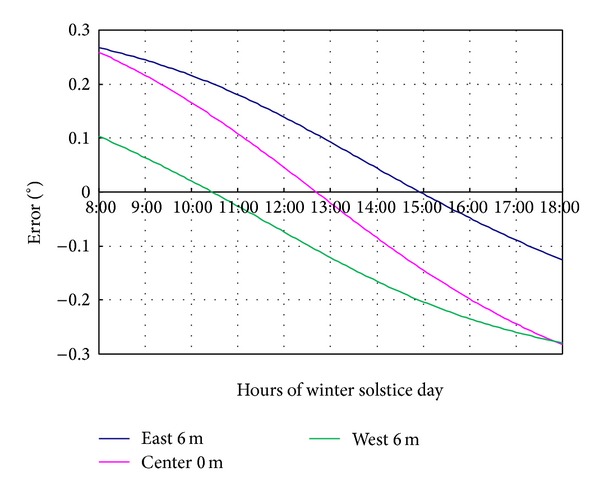
The tracking error caused by eccentric deviation of different column.

Because the rotation axis of reflectors is not located at the geometry center of the mirror, the orbit of incident point on the mirror usually is an ellipse. Meanwhile, reflectors in different columns rotate at different speeds at the same time, which results in different tracking errors.  Figure 3 shows that, at winter solstice, when reflection mirror 1 and reflection mirror in middle column performed 10 cm eccentric distance, at 8:00, the tracking error is 0.26° and the tracking error of reflection mirror 11 is 0.1°. At 18:00, the tracking errors of reflection mirrors 1, 6, and 11 are −0.12°, −0.28°, and −0.27°, and the minimum error happens at 15:00, 12:40, and 10:25, respectively.

**Figure 4:**
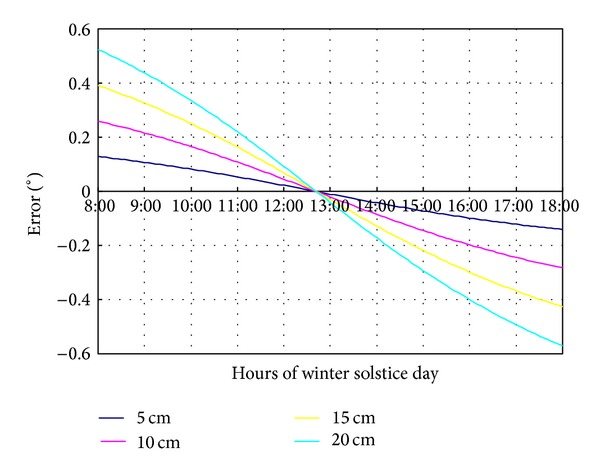
The tracking error caused by eccentric deviation of the middle columns.

Eccentric deviation value has significant effect on the tracking error. It is shown in Figure 4 that the maximum tracking error at winter solstice, caused by different eccentric distances (5, 10, 15, and 20 cm), mainly occurs at 8:00 or 18:00. Nevertheless, the tracking error at around 12:40 is zero, and the ratio of eccentric distance is consistent with the ratio of tracking error.

**Figure 5:**
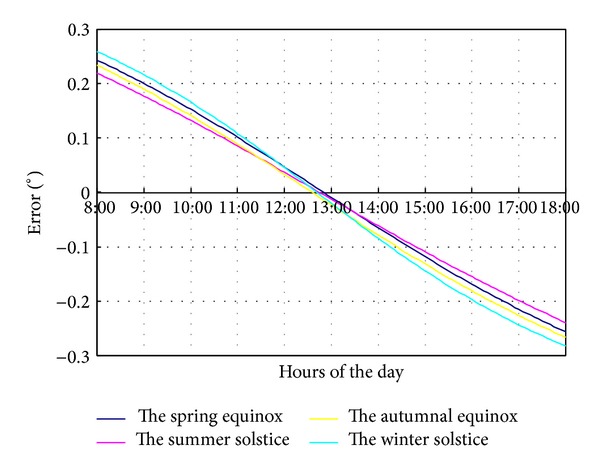
The tracking error on four typical days.

In different dates throughout the year, the tracking error caused by reflector eccentric also changes. As shown in Figure 5, the maximum tracking error of middle column (column 6) with a 10 cm eccentric deviation at the typical dates of vernal equinox, summer solstice, autumnal equinox, and winter solstice happens at 8:00 and 18:00 with about 0.23°. Especially, the maximum tracking error happens at winter solstice.

Because of eccentric influence, different columns and different time lead to different tracking error. On the same day, there is a big error at morning and afternoon but almost no error at a certain moment at noon.

### 3.2. The Influence of North-South Orientation Deviation on Tracking Accuracy

**Figure 6:**
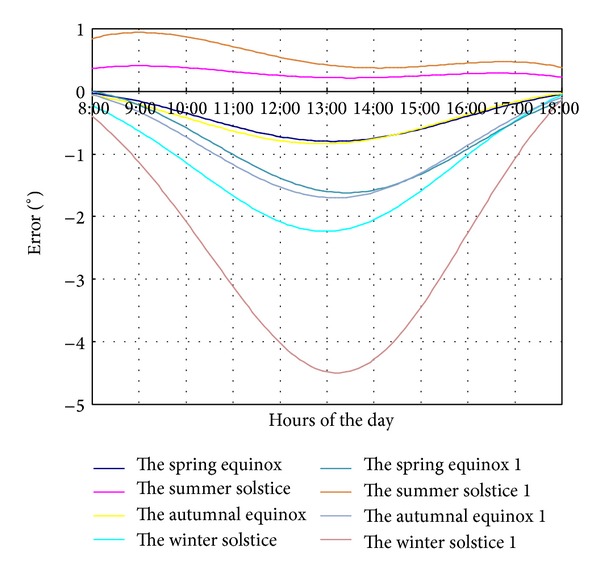
The tracking error caused by north and south direction negative deviation on four typical days.

Because geographic north/south is different from geomagnetic north/south, there exists magnetic declination. The sun location algorithm is based on geographic north and south; thus the truly geographical north/south of solar field is very important for the tracking system. Assuming that southwest is negative deviation for solar field orientation, the influence of north-south orientation deviation on tracking error can be simulated. The variation trend of tracking error for different orientation deviation (5° and 10°) at vernal equinox, summer solstice, autumnal equinox, and winter solstice is shown in Figure 6.

When the orientation axis lays west of 10°, the tracking error curves at winter solstice has the biggest slope. The tracking error increases from 0.66° (8:00) to 4.5° (12:00), then it decreases gradually. At 18:00, the value is around 0.08°.

The tracking errors at vernal equinox and autumnal equinox are much smaller than that at winter solstice. The error value is positive, which means the mirror rotation angle is bigger relative to theoretical tracking angle and the tracking system runs too fast. Nevertheless, at summer solstice, tracking error presents a negative value, which implies that the tracking system is too slow.

The tracking error with an orientation axis deviation of 5° westward, comparing with an orientation axis deviation of 10° deviation, has a similar changing trend, but the value of the former is about half of the latter.

### 3.3. The Influence of Center Height and Center Distance Deviation on Tracking Accuracy

The height of middle column (column 6) and center distance deviation performs no effect on tracking error in each typical day.

When the height of reflectors shows −10 cm and −5 cm deviation, its tracking error is 0.2° and 0.1°, respectively. When the center distance shows −10 cm deviation, its tracking error is 0.4°. In the case of −5 cm deviation, its tracking error is 0.2°.

### 3.4. The Influence of Solar Altitude Angle Deviation on Tracking Accuracy

**Figure 7:**
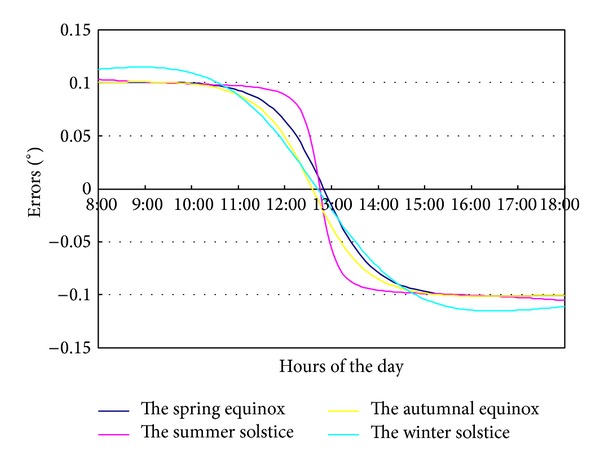
The tracking error of solar altitude 0.2° deviation.

In the Fresnel solar concentrating system, solar altitude angle is one of the key factors for calculating the sun position. It is shown in Figure 7, when the deviation of solar altitude angle is 0.2°, that the tracking error from 8:00 to 11:00 and from 14:00 to 18:00 is about 0.1°. The error is positive in the morning (rotating too fast), while it is negative in the afternoon (rotating too slow). In the three hours from 11:00 to 14:00, the error changes gradually from 0.1° to −0.1°. However, at summer solstice, this change mainly occurs from 12:30 to 13:30.

### 3.5. The Influence of Geographical Latitude-Longitude Deviation on Tracking Accuracy

**Figure 8:**
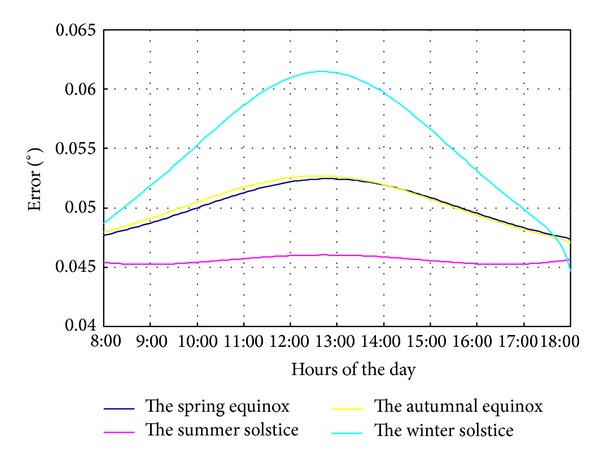
The tracking error of longitude 0.1° deviation.

Owing to the low energy density of solar energy, it usually requires large area for solar power generation; thus the geographical latitude longitudes are different in different area of solar field. As shown in Figure 8, when the longitude has 0.1° deviation at winter solstice, the tracking error increases from 0.047° (at 8:00) to 0.062° (at 12:45) then decreases to 0.045° (at 18:00). The error value and trend at vernal equinox and autumnal equinox are consistent. At summer solstice, the tracking error is almost invariable in the whole day.

**Figure 9:**
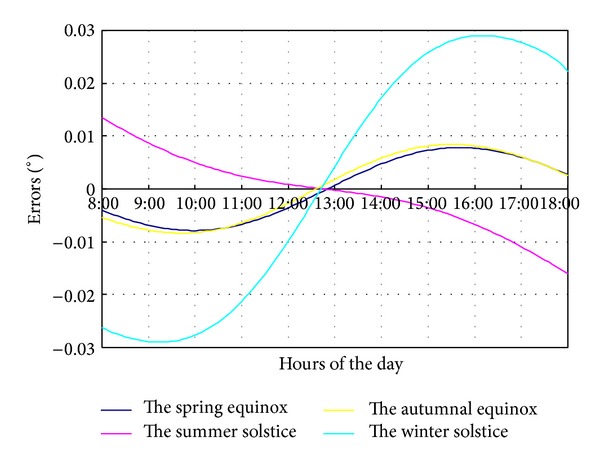
The tracking error of latitude 0.1° deviation.

Figure 9 shows that, when latitude has 0.1° deviation at winter solstice, vernal equinox, and autumnal equinox, the trends of the tracking errors curves are similar. However, the error value at winter solstice is much higher than the other two typical days. Different from other typical day, the error value at summer solstice gradually decreases from +0.013° (at 8:00) to zero then increases to a maximum value of 0.016° at 18:00.

### 3.6. Comparison of All Factors

By calculating the tracking error of middle reflector (column 6) due to the various factors, the comparison results are shown in Table 1.

From Table 1, the order of influence on tracking error is north-south orientation deviation, eccentric deviation, altitude angle deviation, longitude deviation, latitude deviation, center distance, and height deviation successively.

Height deviation almost has no effect on the tracking precision of middle mirror. However, if the height deviation is 10 cm, the tracking error for mirror 1 is 0.2° in typical day, and if the center distance deviation is 10 cm, the error can reach 0.4°.

## 4. Conclusion

There are many influence factors for Fresnel solar tracking error, such as reflector supporting structure, rotation axis position, driver accuracy, tracking software algorithm, deviation of geographic latitude-longitude, and related structure error. When north-south orientation and geographic longitude have deviation, the tracking error is biggest at noon, and it is smaller at morning and afternoon on the same day. When the mirror rotating center, solar altitude, and geographic latitude have deviation, the error value changes from positive to negative, and at a certain moment in the midday, the error is zero.

Tracking error has a great influence on the solar collection efficiency. For Fresnel solar concentrating system, if the error of tracking is 0.1°, the light energy collected by the receiver tubes decreases by 10%. Therefore, improving efficiency and accuracy of automatic tracking system plays an important role in increasing solar collection efficiency and energy generation and decreasing the cost of solar power.

## Figures and Tables

**Table 1:** Effect of the tracing error for the middle mirror.

Influence factor	Eccentric distance	Eccentric distance	Eccentric distance	Eccentric distance
Value	10 cm	10 cm	10 cm	10 cm
Typical data	Winter solstice	Autumnal equinox	Summer solstice	Spring equinox
Maximum error	0.27°	0.23°	0.22°	0.24

Influence factor	Eccentric distance	Eccentric distance	North and south deviation	North and south deviation
Value	5 cm	15 cm	−10°	−10°
Typical data	Winter solstice	Winter solstice	Winter solstice	Spring equinox
Maximum error	0.17°	0.4°	4.5°	1.6°

Influence factor	North and south deviation	Autumnal equinox	Center distance deviation	Height deviation
Value	−10°	−10°	−10 cm	−10 cm
Typical data	Autumnal equinox	Summer solstice	Typical day	Typical day
Maximum error	1.6°	−0.94°	0°	0°

Influence factor	Longitude deviation	Longitude deviation	Longitude deviation	Angle deviation
Value	0.1°	0.1°	0.1°	0.05°
Typical data	Winter solstice	Equinoxes	Summer solstice	Typical day
Maximum error	0.065°	0.053°	0.045°	0.025°

Influence factor	Latitude deviation	Latitude deviation	Latitude deviation	Angle deviation
Value	0.1°	0.1°	0.1°	0.2°
Typical data	Winter solstice	Equinoxes	Summer solstice	Typical day
Maximum error	0.026°	0.008°	0.013°	0.12°
